# Dynamic Visual Cues for Differentiating Mirror and Glass

**DOI:** 10.1038/s41598-018-26720-x

**Published:** 2018-05-30

**Authors:** Hideki Tamura, Hiroshi Higashi, Shigeki Nakauchi

**Affiliations:** 1 Department of Computer Science and Engineering, Toyohashi University of Technology, Toyohashi, Aichi Japan; 2 Japan Society for the Promotion of Science, Chiyoda, Tokyo Japan

## Abstract

Mirror materials (perfect specular surfaces such as polished metal) and glass materials (transparent and refraction media) are quite commonly encountered in everyday life. The human visual system can discriminate these complex distorted images formed by reflection or transmission of the surrounding environment even though they do not intrinsically possess surface colour. In this study, we determined the cues that aid mirror and glass discrimination. From video analysis, we found that glass objects have more opposite motion components relative to the direction of object rotation. Then, we hypothesised a model developed using motion transparency because motion information is not only present on the front side, but also on the rear side of the object surface in the glass material object. In materials judging experiments, we found that human performance with rotating video stimuli is higher than that with static stimuli (simple images). Subsequently, we compared the developed model derived from motion coherency to human rating performance for transparency and specular reflection. The model sufficiently identified the different materials using dynamic information. These results suggest that the visual system relies on dynamic cues that indicate the difference between mirror and glass.

## Introduction

Visual information reaching the human retina potentially contains temporal components due to the motion of objects and/or observers. It has been revealed that several dynamic cues contribute to perception of x-from-motion: e.g., depth, shape, and structure. Although most studies on material perception have focused predominantly on static cues to reveal how the visual system recognises materials, it is highly probable that dynamic visual cues strongly contribute to perception of certain kinds of material, e.g., glossiness^1–8^, transparent flow perception (such as for liquid^9^ and hot air^10^) from image deformation, liquid viscosity perception^11,12^, and stiffness^13,14^.

Mirror and glass have completely different optical properties. They do not intrinsically possess surface colour; thus, humans somehow differentiate mirror and glass by perception of the distorted images formed by reflection or transmission of the surrounding environment. However, the visual features the human visual system uses to achieve this task are still unknown, because the distortion of the reflected/transmitted image is largely and complicatedly dependent on the 3D shape and surroundings of the object. The visual system has constancy for surface properties under natural illumination to some extent^15^, meaning that humans can accurately distinguish various materials under natural illumination via static visual features^16–19^. Therefore, it is natural to assume that the visual system discriminates mirror/glass based on certain visual cues of the distorted images. For example, Kim & Marlow^20^ previously reported an illusion in which a specular reflected object is seen as a refracted and transparent object when it is turned upside-down. They suggested that the reflected/refracted illumination appears right side up for convex mirror surfaces whereas it appears upside down for convex glass surfaces.

This study focuses on mirror and glass materials, because both are, as stated above, quite common in everyday life and easy to discriminate under natural conditions, but both are also determined only based on visual cues existing in the image distorted by the reflection (for mirror materials) or transmission (for glass materials). The type and degree of distortion varies significantly according to the 3D shape of the object, which is generally unknown and should be recovered by the visual system. Therefore, even though it should be difficult for the visual system to discriminate mirror and glass, this is actually quite easy for humans under everyday conditions. We therefore hypothesised that, in addition to static visual cues, certain dynamic cues caused by the motion of the object and/or observers play an important role in the discrimination of these materials.

Although it was previously unknown that dynamic cues are used to directly distinguish mirror and glass, some related cues have been reported for specular reflected or transparent objects. Doerschner *et al*.^1^ have reported that there are characteristic differences between moving matte and shiny objects. They proposed three cues (coverage, divergence, and 3D shape reliability) on which the brain relies to distinguish those objects. In this study, we do not consider matte objects, but aim to distinguish mirror and glass, which can both be regarded as shiny. This is highly challenging, because it is more difficult to distinguish these materials using optic flows only than to distinguish matte and shiny objects (see Fig. S1). Fleming *et al*.^21^ have reported that the distortion field derived from the refractive index of a thick transparent object (glass) determines human perception when judging refractive indices. Although this provides a cue for perceiving glass, this perception might arise because of similar distorted images derived from the specular surface depending on the object’s shape, motion, and surrounding environment. Further, Kawabe *et al*.^9^ have reported that image deformation by some specific spatiotemporal frequencies allows perception of transparent liquid layers. This is one of the cues for perceived transparency for non-rigid objects, but may also be related to rigid glass appearance. These previously proposed cues provided by motion do not directly explain the mechanism used to distinguish mirror and glass.

When a transparent object rotates about its vertical axis, the rear side of the object surface moves in the direction opposite to the object’s rotational direction (e.g., an Ullman cylinder^22^). In other words, dynamic information is not only present on the front side of the object surface, but also on the rear side of the object surface in the glass object. This is because the transparent medium of the glass object transmits the light through the object. This phenomenon is similar to motion transparency^23,24^. Therefore, we hypothesised that a model developed using motion information in the same manner as motion transparency (e.g., refs^25,26^) explains perceptual material discrimination between mirror and glass, based on the results of human psychophysics.

**Figure 1:**
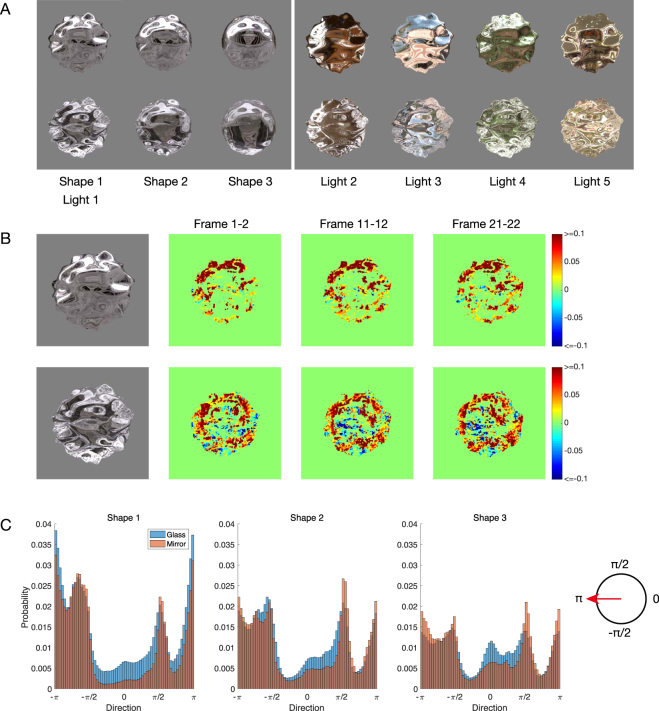
Differences between mirror and glass materials in video stimuli. (**A**) Examples of stimuli (see Movie 1 for the dynamic condition). The top row contains the mirror material objects and the bottom row contains the glass material objects. The left block shows three different shapes under illumination 1 (environment light field). The right block shows five different illuminations with object shape 1. (**B**) Visualisations of motion components in object rotation direction. The top column shows the mirror material objects and the bottom column shows the glass material objects. Colour maps indicate the magnitude of the motion components in each pixel. Red indicates the left direction and blue indicates the right direction. We selected three examples, frames 1–2, 11–12, and 21–22, for shape 1 under illumination 1. (**C**) Histogram indicating the directions motion of the optic flows of mirror and glass materials. The horizontal axis indicates the direction in radians (right is zero and left is pi). The vertical axis indicates the probability of appearance frequency. The optic flows were included for all frames and the five natural illuminations. Note that these components only move in the horizontal direction because the object was rotated around the vertical axis.

To identify the difference between the motion information of the mirror and glass materials, we first computed optic flows using the method previously reported by Lucas and Kanade^27^. Examples are shown in Fig. 1A (see also, Movie 1). A mirror-like material has a specular highlight component on the surface of the object (e.g., polished metal), and glass is a transparent and refractive medium (e.g., ice). The mirror material was defined as a material with perfectly specular reflection on the surface, which is similar to an object having an infinite refractive index. The glass material was defined as a material with a transparent and refractive medium, with a refractive index of 1.5, to be similar to common glass (see Stimuli). The stimuli comprised 60 video frames rendered with three different shapes under five real-world illuminations^28,29^, and an object rotating about the vertical axis. A colour map indicating the magnitudes of the horizontal motion components in each pixel is shown in Fig. 1B and Movie 2. In the mirror object, brighter luminance areas, such as specular highlights and the contour configuring the object’s shape, predominantly moved in the object’s rotational direction. In the glass object, the components described above and additional opposite motion components were present around the centre of the object. Here, we quantitatively express this difference using a histogram (Fig. 1C). The histogram indicates the directions of motion of the optic flows of the mirror and glass materials. Figure 1C shows that there are peaks towards the right (0 rad) in the glass materials in each shape. The glass material has more components towards the right than the mirror material. This is because it is a transparent object and opposite direction components exist on the rear side of the object surface such as motion transparency.

From these observations, the visual system would use the distinctive motion information to distinguish mirror and glass. If so, perceptual material discrimination performance with motion could be better than without motion. In experiment 1, we compare the performances with/without motion. Then, in experiment 2, we collect human rating data for perception of transparent and specular reflection to propose a model explaining the human perception in this task. Finally, in experiment 3 and experiment 4, we validate the model by excluding motion transparency and static cue.

## Results

### Perceptual Discrimination between Mirror and Glass

To test whether the visual system uses distinctive motion information to distinguish mirror and glass, we compared the performance of two presenting conditions: static and dynamic. Under the static condition, the observer was presented with a single image frame, randomly extracted from the video. The dynamic condition was simply presented in video form. These conditions were defined as ‘original’, and an additional condition was defined as ‘upside-down’ stimuli, in which images were rotated by 180°; this operation was performed in order to measure the amount of additional information provided by static cues^20^. Both original and upside-down stimuli were intermingled in one block. One stimulus (60 frames) was displayed on a liquid crystal display (LCD) monitor for 1000 ms (i.e. the frame rate was 60 frame/s), and observers were asked to state their opinion on which material (mirror or glass) was being observed in a yes/no task paradigm. The observer performance was defined as the average of the percentage of correct answers for the mirror and glass materials (see Procedure & Task).

**Figure 2:**
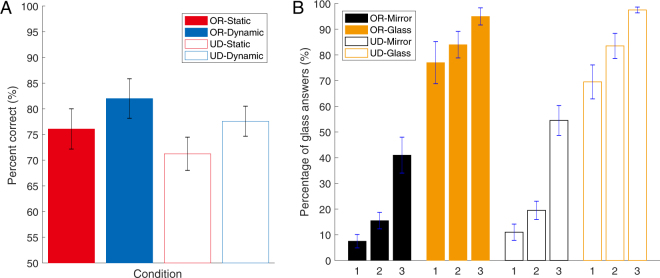
Perceptual material discrimination between mirror and glass. (**A**) Results for perceptual material discrimination under two presenting conditions, along with the rotating conditions. The horizontal axis indicates each condition combined with the rotating and presenting conditions. The vertical axis indicates the percentage of correct answers. ‘OR’ signifies original, and ‘UD’ signifies upside-down stimuli. Averages and standard errors among observers were obtained. The error bars represent the standard error of the mean across all ten observers. (**B**) Percentage of glass answers for different shapes. The horizontal axis indicates the shape number and the vertical axis indicates the percentage of glass answers. Different numbers along the horizontal axis indicate different shapes. The symbols are the same as in **A**.

Figure 2A shows the performance for each condition. We performed two-way repeated-measures analysis of variance (ANOVA) for the presenting condition and the rotating condition. The main effect of the presenting condition was significant (*F*(1, 9) = 18.311, *p* < 0.005) and indicated that the performance under the dynamic condition was higher than that under the static conditions. The main effect of the rotating condition was significant (*F*(1, 9) = 10.018, *p* < 0.01), meaning that turning the image upside-down decreased the performance of perceptual material discrimination. There was no significant interaction (*F*(1, 9) = 0.048, *p* = 0.832).

The dynamic condition exhibited higher performance, in support of our hypothesis. Under this condition, the visual system acquired consecutive images as rich three-dimensional structural information from the video, as in ref.^22^, for example. The three-dimensional information gives a cue to albedo estimation on the surface^30,31^. This result suggests that there is a cue for not only albedo estimation, but also perceptual material discrimination between mirror and glass materials, even those with complex optical features on their surface.

The performance for the upside-down stimuli was significantly lower than that for the original stimuli. One reason for this is that turning an image upside-down decreases a cue derived from ‘the light-from-above prior’, i.e. the assumption that light simply comes from above one’s head^32–35^. These results suggest that both the dynamic cue derived from the video stimuli containing consecutive images and the static cue derived from the image itself contribute to perceptual material discrimination.

Then, to determine the motion information, we confirmed a tendency associated with the percentage of glass answers in each shape and the rotating conditions under the dynamic condition (Fig. 2B). Specifically, the percentage of glass answers increases depending on the bumpiness of the shape, regardless of the material. This suggests that the more spherical an object is, the more easily it is perceived as being made of glass material, as shape 1 in Fig. 1 has the bumpiest surface, whereas shape 3 has the least bumpy surface.

We also included a ‘shuffle’ condition to examine whether the increasing performance was not simply caused by the number of viewpoints. Although the shuffle condition provided image information from various viewpoints, similar to the dynamic condition, its performance was almost same as the static condition. Simultaneously, we tested the effect of changing the luminance polarity to measure the amount of information provided by static cues derived from the natural environment, similar to the upside-down condition. This operation had the same tendency as that of turning upside-down (see supplementary experiment).

### Model Development and Performance

**Figure 3:**
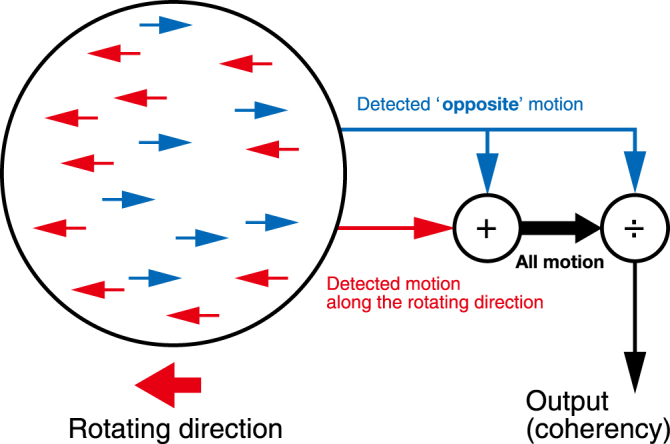
Model description.

Our findings (Fig. 1B,C) suggest that it is possible for the simple quantified index to express perceptual material discrimination between the mirror and glass materials. We defined a quantified index *k* as the motion ratio between the direction of object rotation and its opposite direction based on the relationship between motion coherency and behavioural performance^26^ (see Quantification for the dynamic cue). Figure 3 illustrates the model. We assumed that the visual system detects two kinds of motion: (1) motion in the object rotation direction and (2) motion in its opposite direction, and we estimated the ratio of the opposite motion in all motions.

It is possible to develop a model that can explain not only two specific materials (mirror and glass), but also a general material with a smooth surface depending on the refractive index. To achieve this goal, we collected the rating data for specular reflection and transparency as perceived by the human observers. We assumed a general material with a smooth surface and material property changing with the refractive index. Then, we created images of an object composed of a material with an arbitrary refractive index via image morphing, using the mirror and glass materials (see Stimuli). Finally, we prepared nine different materials as stimuli with nine different refractive indexes (1.33, 1.5, 1.7, 2.42, 5, 10, 20, 50, and infinite). The stimuli were then presented under the dynamic condition (i.e. as video) and the human observers rated the materials on a seven-point scale from perceived specular reflection to perceived transparency. The other conditions were the same as in the experiment for perceptual material discrimination.

**Figure 4:**
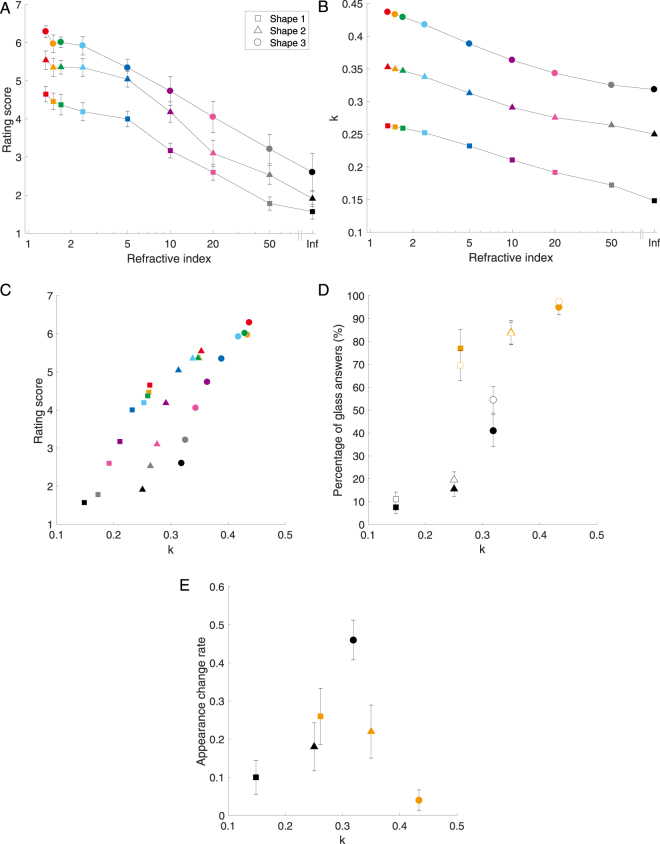
Developed model and its performance. (**A**) Rating score of the perceived specular reflection and transparency. The horizontal axis indicates the refractive index of the stimuli on a log scale. The vertical axis indicates the rating score on a seven-point rating system, where perceived specular reflection is one and transparency is seven. Averages and standard errors among observers were obtained. The error bars represent the standard error of the mean across all ten observers. (**B**) Model output. The horizontal axis is the same as in **A**. The vertical axis indicates the model output *k*. (**C**) Correlation between model output and ratings given by human observers. (**D**) Correlation between the model output *k* and the percentage of glass answers for original and upside-down stimuli under dynamic conditions. The horizontal axis indicates the model output *k*. The vertical axis indicates the percentage of glass answers; this axis is the same as in Fig. 2B. The filled and open symbols indicate the original and upside-down stimuli, respectively. Averages and standard errors among observers were obtained. The error bars represent the standard error of the mean across all ten observers. (**E**) The appearance change rate. The horizontal axis is the same as in (**C** and **D**). The vertical axis shows the rate indicating how much the material appearance changes. In (**B**–**E**) the symbols are the same as in **A**.

Figure 4A shows the rating score obtained for each condition. We performed two-way repeated-measures ANOVA for the refractive index and shapes. The main effect of the refractive indexes was significant (*F*(1.665, 14.989) = 58.57, *p* < 0.001). The material with the same refractive index (infinite) as the mirror material was rated significantly lower than others; in other words, it was perceived as having the most specular reflection among all the different refractive index materials (multiple comparison test). The main effect of the shapes was significant (*F*(1.263, 11.366) = 50.562, *p* < 0.001). The ratings for the shapes were significantly different (multiple comparison test; shape 1 vs. shape 2 (*p* < 0.01); shape 1 vs. shape 3 (*p* < 0.001); shape 2 vs. shape 3 (*p* < 0.001)) and shape 3 obtained the highest rating; in other words, it was perceived as being the most transparent object. There was no significant interaction (*F*(16, 144) = 1.100, *p* = 0.361). Note that we described adjusted degrees-of-freedom using Greenhouse-Geisser correction when the assumption for sphericity was not in effect (Mauchly’s test of sphericity).

The ratings changed depending on the materials, which physically changed in accordance with the refractive index. This means that the visual system can appropriately rate stimuli simulated by image morphing. In addition, there was a tendency to perceive less bumpy objects as being more transparent. Thus, the perception of transparency changes for three-dimensional shapes, much like the perception of glossiness^36^. The difference in rating score between shape 1 (most bumpy) and shape 3 (least bumpy) was 1.48 on average. This suggests that the object shape easily changes human perception of a material even if it has the same refractive index.

Figure 4B shows the relationship between the refractive indexes and the output score *k* of the model. As in Fig. 4A, it expresses a nonlinear function that indicates the relationship between the refractive indexes and the rating given by the human observers. Further, the model output well explains the rating via the refractive index shown in Fig. 4C. Note that these results are significantly correlated (*r* = 0.83, *p* < 0.001). We also performed a two-way ANOVA without replication of the model output. The main effect of the refractive indexes was significant (*F*(8, 16) = 175.15, *p* < 0.001). The main effect of the shapes was also significant (*F*(2, 16) = 1854.03, *p* < 0.001). These statistical features of the model output are consistent with the statistical features of the ratings given by the human observers. Therefore, this model offers an explanation, i.e. that the visual system uses dynamic information for material perception of a rotating object. Note that the correlation between these results is not perfect, because some factors that are not affected by either the refractive indexes or the shapes remain.

Moreover, the model can also explain the results of perceptual material discrimination. Figure 4D shows the relationship between the model output, which is used instead of the shape conditions, and the percentage of glass answers. This indicates the extent to which the model estimates the observer performance for perceptual material discrimination. If the dynamic cue contributes to this material perception, the model output and the percentage of glass answers should be connected in a correlative relationship. Note that our premise is that the relationship between the rating dimensions from the perceived specular reflection to transparency and the percentage of glass answers obtained in a yes/no task paradigm is a monotonically increasing function. In the original case, although the percentage of glass answers increased with the model output, there is a separation between the mirror and glass materials (in Fig. 4D, the filled symbols). This suggests that the dynamic information and other factors contribute to the material perception. The question now becomes the following: which static cues contribute to the distinction between mirror and glass materials? Considering that these cues exist among the original stimuli but not among the upside-down stimuli, one possibility is the luminance distribution along the vertical direction of the stimuli, because the effect derives from ‘the light-from-above prior’^32–35^. These results suggest that the visual system naturally hypothesises that the light is located above the observer’s head. We thus speculate that the other factor is a static cue from the image.

In contrast, when we rotated the image by 180°, the model output and percentage of glass answers were significantly and more highly correlated (*r* = 0.88, *p* < 0.05) (in Fig. 4D, open symbols). This correlation coefficient was greater than that obtained with the original stimuli. Although both the original and upside-down stimuli had the static and dynamic cues, the visual system could not sufficiently use the static cue in the upside-down stimuli because the images were rotated, which inhibits the static cue. Therefore, the visual system was basically more dependent on the dynamic cues.

In addition, turning upside-down seems to more strongly affect to our perception when the *k* value was medium (in this case, at around 0.3). To quantify the effect of turning upside-down, we focused on the change of material appearance (mirror-to-glass or glass-to-mirror) by turning upside-down to manipulate the static cue and calculated the ratio of the appearance change for various *k* values. Figure 4E shows the appearance change ratio significantly varied depending on the *k* value (*F*(5, 45) = 10.281, *p* < 0.001; one-way repeated-measures ANOVA). A multiple comparison test showed that the change rate of the mirror of shape 3 (black circle) was significantly higher than that of the mirrors of shapes 1 and 2, and the glass of shape 3 (*p* < 0.05). These results suggest that the effect of the dynamic cue is limited, i.e. the visual system relies more on the static cue when the dynamic cue is ambiguous for distinguishing mirror and glass.

### Model validation

Our model predicts that glass objects may be misperceived as mirror surfaces if the glass generates little or no motion transparency. To test this prediction, we attempted to reduce the amount of motion transparency that glass objects generate by rendering the glass as a purely transmittance component that only refracts light and does not generate any specular reflection.

**Figure 5:**
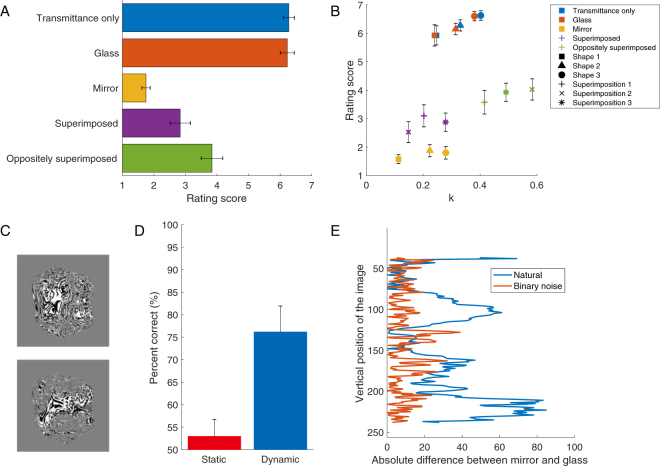
Validation of the proposed model with new stimuli. (**A**) Rating scores of perceived specular reflection and transparency for new stimuli. The horizontal axis indicates the rating score corresponding to the vertical axis of Fig. 4A. The vertical axis indicates the stimulus conditions. The error bars represent the standard error of the mean across all ten observers. (**B**) Relationship between model output and rating score. (**C**) Stimuli rendered in random binary noise illumination (top: mirror, bottom: glass). See also, Movie 4A and B. (**D**) Result of perceptual material discrimination using the binary noise condition. The horizontal axis indicates each condition. The vertical axis indicates the percentage of correct answers. Averages and standard errors among observers were obtained. The error bars represent the standard error of the mean across all seven observers. (**E**) Different luminance distributions between original and the binary noise conditions. The horizontal axis indicates the absolute differences of the image pixel intensities (luminance) between mirror and glass. The pixel intensities were averaged along each row in the image, excluding the background. The vertical axis indicates the vertical position of the image.

We generated three stimuli: (1) glass only, possessing the transmittance component of the light (‘transmittance only’, Movie 3A) and created through image morphing (see Stimuli); images created by superimposing two different shape mirrors images in the (2) same (‘superimposed’, Movie 3B) and (3) opposite directions (‘oppositely superimposed’, Movie 3C). The last two also changed the amount of motion transparency. Figure 5A shows the rating scores for five conditions including actual mirror and glass. We performed one-way repeated-measures ANOVA for the stimulus conditions. The main effect for the stimulus conditions was significant (*F*(1.962, 17.656) = 68.842, *p* < 0.001, adjusted degree-of-freedom using Greenhouse-Geisser correction). We predicted that the ‘transmittance only’ condition would be perceived as being more transparent than the glass, because the reflectance component of the former is eliminated, and its *k* value increases according to the model. However, there was no significant difference (multiple comparison test; transmittance only vs. glass (*p* > 0.05)), suggesting that the transmittance component mainly provides us with transparency perception. The superimposed and oppositely superimposed conditions were perceived as being more transparent than the mirror (multiple comparison test; mirror vs. superimposed (*p* < 0.001); mirror vs. oppositely superimposed (*p* < 0.001)). We speculate that the superimposition of two different shapes provides humans with an impression of a transparent layer on the surface. The oppositely superimposed condition additionally increased the opposite motion relative to the rotation direction (the model output *k*, see Fig. 5B). This was not the same rating as the glass, because the pixel information derived from the mirror reduced the perceived transparency. If we could naturally match two different shapes on a pixel-by-pixel basis, a clearer glass appearance would be obtained. Then, the relationship between the *k* value and the rating score would more generally support the model.

Even if the rotating stimuli in which static cues were maximally eliminated, motion transparency would still provide sufficient cues to distinguish the materials. To test this, we rendered new stimuli (Fig. 5C, see also Movie 4A and B) in a random binary noise illumination using more complex shaped objects based on our proposed model. We tested them using a material judgement test. Figure 5D shows that the dynamic condition was significantly higher than the static condition (*t*(12) = 3.399, *p* < 0.01, t-test), supporting our findings. This result suggests that the dynamic cue is informative for the distinction of materials, even without a simple static cue. Moreover, we found a tendency that the luminance distribution in the vertical axis of the binary noise condition was almost flat and the absolute difference between mirror and glass was smaller than that for the natural illumination (Fig. 5E). In the real world, we naturally exhibit a luminance bias, i.e. the upper area of the object surface is brighter, and the bottom area of the object surface is darker because the lights from sources such as the sun and illumination lamps tend to come from above. However, in the binary noise condition, this bias does not exist, and the visual system cannot rely on this cue. We speculate that measuring this kind of difference provides us with information on how the visual system is sensitive to the static cue.

## Discussion

Previous studies on surface properties focussed on highly reflective surfaces and included dynamic information^1–8^. It has also been suggested that three kinds of cues from optic flows can distinguish the surface property (matte or shiny) of an object^1^. Both the mirror and glass materials in our study were shiny objects and had similar features to identify the shiny surface. In addition, our results reveal that the optic flows can identify whether the material of a rotating object is mirror, glass, or its medial materials depending on the refractive index. We suggest that this dynamic cue from the video can somehow be detected by the visual system, for example, through perception of motion transparency, and be used for perception of smooth surface materials and distinction between mirror and glass.

In this study, the visual system could distinguish between materials under the static condition to an extent, because it can use a distorted image reflected from surrounding illumination on the surface of the mirror object^37^, and a refraction image derived from the shape of thick transparent objects such as glass^21^. A recent study reported that a specular object and a transparent object perceptually change each other by vertically reversing, and suggested that the direction of illumination affects this phenomenon^20^. In ‘the light-from-above prior’, for the mirror object, the upper area of the object surface becomes brighter because the light reflects off the object. Likewise, for a glass object, the bottom area of the object surface tends to become brighter because the light is transmitted through the object. Our results suggest that the visual system uses such a simple static cue for perceptual material discrimination between mirror and glass materials. Further, image statistics could be given as the other static cue in this case, because the visual system can estimate a surface property (glossiness) on the object using those statistics^38^ (but subsequent works have questioned the idea that those statistics play a causal role in material perception; see refs^39,40^). The stimuli for our study were comprised of mirror and glass materials, where both had a specular highlight component and their surfaces were glossy. Therefore, we speculated that a cue from the image statistics was not the only contribution to the material distinction, and that other luminance information, such as the luminance distribution along the vertical direction of the stimuli, also contributed to this distinction.

An image on the surface of mirror object is more clearly visible than that on glass because the surrounding illumination is directly reflected by the surface. By contrast, an image on the surface of a glass object has complex patterns due to the distortion field^21^. We premised that these midlevel features distinguish the reflected image from the surrounding illumination or the refracted image through the background. The static cue surely has an influence on overwriting the dynamic cue. We suggest that the visual system automatically shifts its focus to use only one cue or multiple cues in various contexts.

We found that the magnitude of the object’s bumpiness provides an effect to aid perception of specular and transparent materials (Figs 2B and 4A). A smooth surface tends to be perceived as glass, because it is easy to track the motion on the object’s rear surface. In contrast, a bumpy surface tends to be perceived as specular, because there are enough feature points to track the motion on the object’s front surface. Some material appearances change largely depending on bumpiness (e.g. specularity^41,42^ and translucency^43^). Thus, we speculate that the ability to track the object motion aids distinction between mirror and glass.

Some observers reported perceiving liquid in the rotating glass material object, especially in shape 3 (lowest bumpiness). Almost all liquids are transparent or translucent, and recent studies have reported the importance of liquid perception for the visual system^9–12,44^. In our study, although the glass material object was definitely rigid and solid, the visual system perceived a non-rigid object like water, because the visual system distinguishes non-rigid object shapes from the motion cue^45^. This study was mainly aimed at rigid objects only; however, it is necessary to discover how the visual system distinguishes between non-rigid specular and transparent objects such as mercury and water.

From neuroscience and neurophysiological perspectives, our glossiness perception^46,47^ and material perception^16,48^ are represented in the inferior temporal (IT) cortex through the ventral pathway. The structures determined from motion^49,50^ and motion transparency are represented in the middle temporal area (MT)^25,51^ and the fundus of the superior temporal sulcus (FST)^52^ through the dorsal pathway. It is possible that both processes are in parallel or provide mutual feedback to each other, raising the recognition level. We suggest that examination of the relationship between them will provide further insight into the perception of smooth surface materials by dynamic cues.

In-depth understanding of the material perception performed by the visual system can be obtained by considering not only the static cue from the image, but also the dynamic cue, because the findings of this study provide one piece of evidence to explain this mechanism. Our finding can also explain perception of any motions other than rotation. In future work, we would like to study not only smooth surface objects such as mirror and glass materials, but also objects with matte surfaces such as wood, fabric, and other materials, or a mixture of materials.

## Methods

### Observers

Experiment 1 (perceptual material discrimination between mirror and glass materials): Ten naïve observers participated in this experiment. Their ages ranged from 21 to 25 years (average 22.4 ± 1.4 years).

Experiment 2 (rating for perception of transparent and specular surface): Ten naïve observers, who had not participated in experiment 1, participated in this experiment. Their ages ranged from 22 to 25 years (average 23.2 ± 1.1 years).

Experiment 3 (rating using edited videos): Ten naïve observers participated in this experiment. Their ages ranged from 23 to 26 years (average 24.2 ± 1.1 years).

Experiment 4 (perceptual material discrimination using binary noise stimuli): Ten naïve observers participated in this experiment. Three observers were excluded because their performance for the static condition was equal to or higher than that for the dynamic condition and quite different from the others. Thus, the final sample consisted of seven observers aged from 23 to 26 years (average 24.1 ± 1.1 years).

All observers had normal or corrected-to-normal acuity. All experimental protocols were approved by the institutional review board of Toyohashi University of Technology on the use of humans in experiments. Informed consent was obtained from all observers and all methods were performed in accordance with the approved guidelines and regulations of the review board.

### Apparatus

Stimuli were displayed on a calibrated 32-inch LCD (Display++, Cambridge Research Systems) with 1920 × 1080 pixel resolution and a 60-Hz refresh rate. Stimulus presentation was controlled by MATLAB using Psychtoolbox 3.0^53–55^. While they observed the stimuli, each observer was seated on a chair facing the display in a dark booth, with their head secured on a chin rest to maintain a constant distance (57 cm).

### Stimuli

#### [Modelling]

The stimuli were modelled using Blender 2.77. First, the object was created as a UV sphere with 10 segments and 10 rings. Then, the subdivide function of the mesh tool was used with the number of cuts set to one, fractal set to 15, and along the normal set to one as parameters for subdividing shape 1. For shape 2, these parameters were 1, 10, and 1, respectively; and for shape 3, 1, 5, and 1, respectively. Then, a subdivision surface was added to these objects, with two views and two renderers using the modifier tool. Finally, the object was set as having a smooth surface using the shading smooth function. The specific modelling procedures were as described above and the other procedures retained the default parameters.

#### [Rendering]

The reflectance and transmittance of smooth-surface materials are theoretically defined by the refractive index of the material^56^. For example, the refractive index of common glass is 1.5 and its reflectance and transmittance are 0.04 and 0.96, respectively. Therefore, most of the incoming light passes through the object and we perceive the object as being made of glass. As the refractive index increases, so does its reflectance (see Fig. S2). Moreover, when the refractive index approaches infinity, all components of light are reflected. Some metals, such as silver and aluminium, have a complex refractive index and their reflectance exceeds 0.9. Therefore, the light is almost perfectly reflected by the material surface and humans perceive it as a polished metal such as a mirror. All objects were rendered using the Mitsuba renderer^57^. For the natural illuminations, we used two light fields, called ‘Uffizi Gallery’ and ‘Dining room of the Ennis-Brown House’, from the High-Resolution Light Probe Image Gallery^29^, and three light fields, called ‘Whiteley shopping centre’, ‘Bolderwood forest’, and ‘Piazza café’, from the Southampton-York Natural Scenes (SYNS) dataset^28^. For the binary noise illumination, we set a random binary noise image as an environment illumination. The object was set on the default position in the scene. The camera was located at five units from the object. For the mirror material properties, the bidirectional scattering distribution function (BSDF) parameter was a conductor and the surface was set to 100% specular reflection. For the glass material properties, the BSDF parameter was a dielectric, the internal refraction index was 1.5 (as with common glass), and the external refraction index was 1.0 (as in air). With the camera fixed, the object was rotated about the vertical axis by 0.5° per frame. We rendered 60 frames per stimulus. The sampling count was 512 per pixel with a low-discrepancy sampler. The reconstruction filter was set as Gaussian and each output frame was a 512 × 512 image. Finally, all images were resized to 256 × 256, and a 1000-ms video containing 60 frames was created. Stimuli for the ‘original’ condition were created using the above procedures. Then, stimuli for the ‘upside-down’ condition were finally obtained via an operation that rotated the image by 180°.

#### [Image morphing for rating experiment]

When light is input from a medium with refractive index *n*_*i*_ to another medium with refractive index *n*_*t*_, the reflectance *R* and transmittance *T* of the output light are expressed as follows:

1 $$R={(\frac{{n}_{t}-{n}_{i}}{{n}_{t}+{n}_{i}})}^{2}$$

2 $$T=\frac{4{n}_{t}{n}_{i}}{{({n}_{t}+{n}_{i})}^{2}}$$

For example, when the light is input from air, which has a refractive index of one (*n*_*i*_ = 1), to a medium with refractive index 1.5 (*n*_*t*_ = 1.5), such as common glass, *R* and *T* are 0.04 and 0.96, respectively. A target image that has an arbitrary refractive index was made using (a) a perfect reflectance image, (b) a perfect transmittance image, (c) the target reflectance, and (d) the target transmittance. The perfect reflectance image was simply an image of the mirror material. The perfect transmittance image was created using the following steps: (1) the image of the mirror was multiplied by 0.04 and the resultant image was outputted; (2) the image of the glass material was subtracted from the image in (1) and the output was divided by 0.96. Finally, the sum of the multiplication products of (a) and (b) and (c) and (d) was used as the target image. This image morphing was performed for the hi-dynamic range components and the image was processed using gamma curve correction (gamma = 2.2). See also Fig. S4.

### Procedure and Task

#### [Experiment 1]

At the beginning of the experiment, a grey background (33.7 cd/m^2^) and fixation point were displayed, which remained throughout the experiment. After a 1-min adaptation period, the first trial was started by pressing a key. A target stimulus was randomly presented for 1000 ms at a 60-Hz refresh rate. An image was presented as the static stimulus for the static condition.

The image was one frame selected once from four frames (frame numbers 1, 16, 31, and 46) extracted from the video (60 frames). The performance for the static condition was the average result for those four frames. A video was presented as the dynamic stimulus for the dynamic condition. To cancel any effect of rotation direction, we prepared trials in which the object rotated leftward and rightward about the vertical axis; these rotations were each included twice in the stimulus conditions. The performance of the dynamic condition was the average result for those four conditions (two trials × two directions). The observer responded by pressing a key on a numerical keyboard to indicate mirror or glass material. The experiment was composed of 480 trials (two materials × three shapes × five illuminations × two rotations × eight present conditions), and all trials were randomly ordered.

As the control, we prepared the shuffle condition, which had four frames as described above, but it randomly presented four frames (frame numbers 1, 16, 31, and 46) in 15 frames as a cut-off animation. For this condition, the observers could obtain image information from various viewpoints, similar to the dynamic condition, but virtually no dynamic information (such as rotational motion) was provided (see supplementary experiment).

#### [Experiment 2]

The procedure was the same as in experiment 1, except the yes/no task was replaced with the seven-point rating task. All stimuli were presented under the dynamic conditions. The experiment was composed of 270 trials (two trials × nine materials × three shapes × five illuminations), and all trials were randomly ordered.

#### [Experiment 3]

The procedure was the same as in experiment 2. The experiment was composed of 60 trials (four trials × five conditions × three shapes), and all trials were randomly ordered.

#### [Experiment 4]

The procedure was the same as in experiment 1, except the upside-down condition was excluded, and the binary noise condition was included. The experiment was composed of 96 trials (two materials × three shapes × two illuminations × eight present conditions), and all trials were randomly ordered.

### Video Analysis

We estimated the optic flows from each pair of frames of the video stimuli using the Lucas-Kanade method^27^ in the MATLAB Computer Vision Toolbox. For preprocessing, the stimuli were sharpened using image sharpening processing. The optic flows in the vicinity along the object contour were excluded, because the effect of the optic flows along an object contour inhibits true temporal deformation information on the object surface.

### Quantification for Dynamic Cue (Model Output)

The dynamic cue was defined as the ratio between the positive and negative value motion components along the object rotation direction. In this case, all stimuli were horizontally rotated, and the object rotation direction was the *x* direction. The ratio *k*_*t*_ between the *t* th frame and the *t* + 1 th frame was defined as described below. The horizontal components of the optic flows *v*_*x*_(*i*, *j*, *t*) between two frames (*t* and *t* + 1) of the material were used to calculate *k*. The step function *f* was defined with parameter *a*, which is related to the threshold of the step function. We set *a* = 0.001. The stimulus had 60 frames and the optic flows were produced as 59 frames (*T* = 59). Finally, the dynamic cue of one stimulus, in other words, the model output *k*, was defined as the average of the ratios of all frames.

3 $$k=\frac{1}{T}\sum _{t=1}^{T}{k}_{t}$$

4 $${k}_{t}=\frac{{\sum }_{i,j}f(\,-\,{v}_{x}(i,j,t);a)}{{\sum }_{i,j}f({v}_{x}(i,j,t);a)+{\sum }_{i,j}f(\,-\,{v}_{x}(i,j,t);a)}$$

5 $$f(x;a)=\{\begin{array}{c}1\,({\rm{if}}\,x > a)\\ 0\,({\rm{if}}\,x\le a)\end{array}$$

### Data availability

The datasets generated during and/or analysed during the current study are available from the corresponding author on reasonable request.

## Electronic supplementary material

Movie 1

Movie 2

Movie 3A

Movie 3B

Movie 3C

Movie 4A

Movie 4B

Supplementary Information
