# Supplementary material for: Dynamic Visual Cues for Differentiating Mirror and Glass
Source: Sci Rep. 2018 May 30;8:8403. doi: 10.1038/s41598-018-26720-x (PMC5976772; doi:10.1038/s41598-018-26720-x)
Supplement: Supplementary file 8 — Supplementary Information [file 41598_2018_26720_MOESM8_ESM.pdf]

Supplementary Information

Dynamic Visual Cues for Differentiating Mirror and Glass

Hideki Tamura[1,2,*], Hiroshi Higashi[1], Shigeki Nakauchi[1]

[1]Department of Computer Science and Engineering, Toyohashi University of Technology, Toyohashi, Aichi, Japan

[2]Japan Society for Promotion of Sciences, Chiyoda, Tokyo, Japan

*Corresponding author

Email: tamura13@vpac.cs.tut.ac.jp

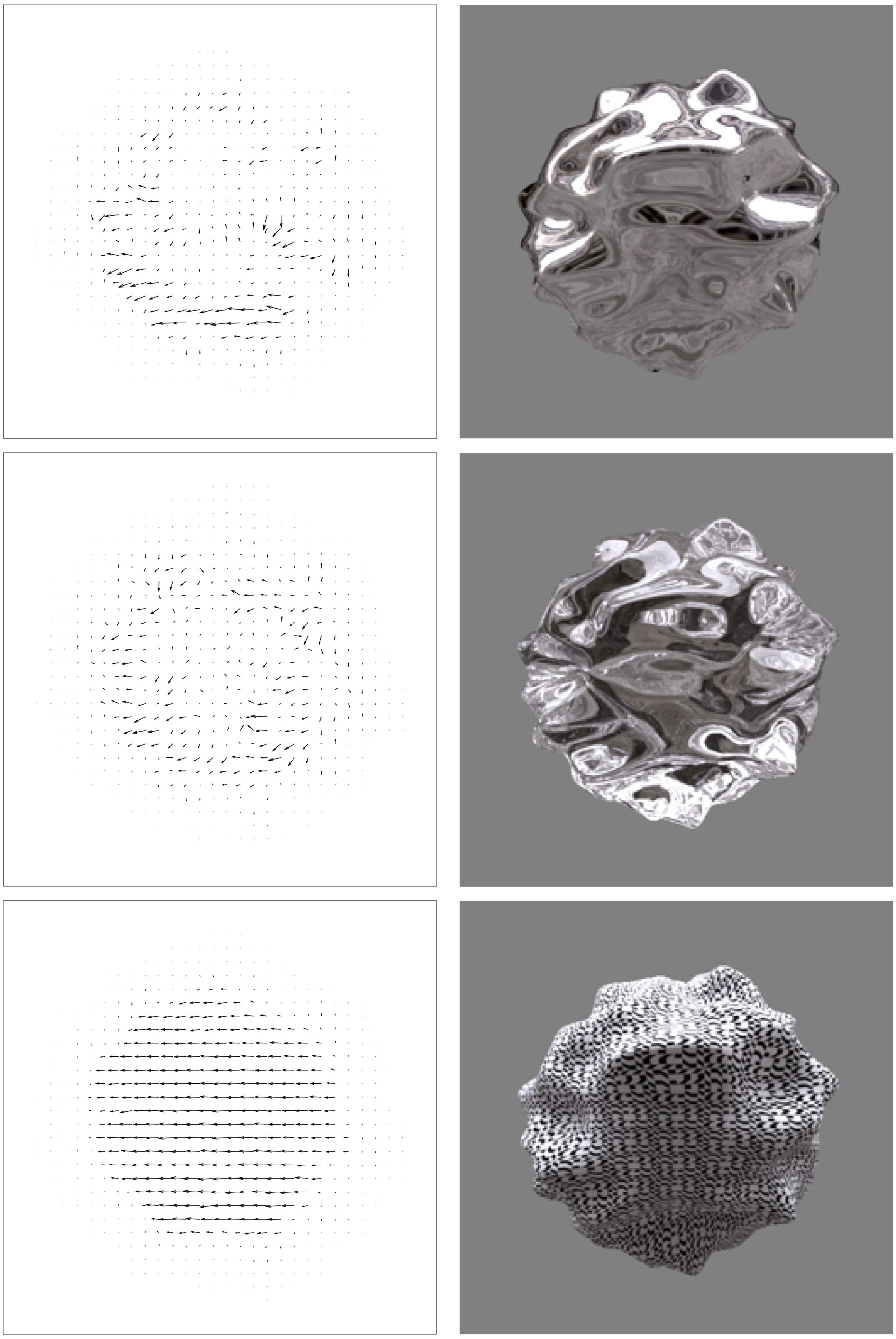

Figure S1. Optic flows in different materials.

The optic flows (in the left column) were generated from between the first frame (in the right column) and the second frames of the videos, which featured three different materials, i.e., mirror, glass, and matte (textured).

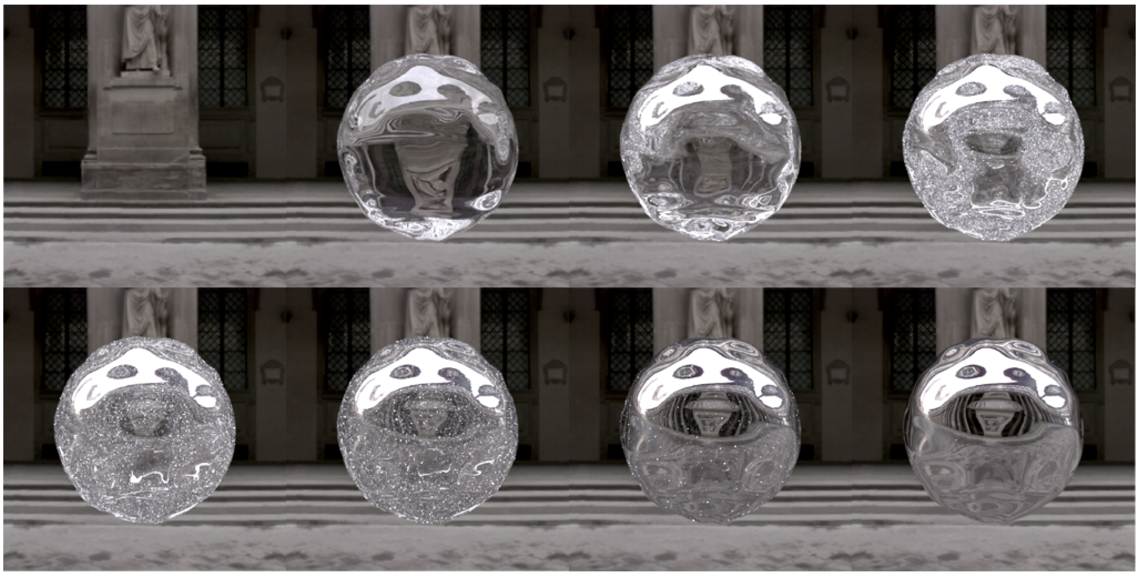

Figure S2. Material appearance with changing refractive index.

Eight glass objects with different refractive indices (1.0, 1.5, 2.5, 5.0, 7.5, 10, 20, and 50 from the top left to the bottom right) are shown.

Supplementary experiment

Observers: Ten naïve observers participated in this experiment. One observer was excluded from analysis because their performance was almost equal to the chance level (50%) and quite different from the others. Thus, the final sample consisted of nine observers ranging in age from 23 to 26 years (average 24.2 ± 1.2 years).

Procedure and Task: The procedure was the same as in experiment 1, except the shuffle and the colour inversion conditions were included. The shuffle condition was set as the control condition. It was comprised of four frames extracted from the video presented in a random sequence, like a cut-off animation (see also Stimuli). All three presenting conditions (static, dynamic, and shuffle) were defined as 'natural'. An additional condition was defined as 'colour inversion' stimuli, in which colours were inverted so that positive values became negative in RGB colour space. This condition was created to measure the amount of information provided by static cues derived from the luminance polarity of the natural environment. Both natural and colour inversion stimuli were intermingled in one block. The experiment was composed of 720 trials (two materials × three shapes × five illuminations × two naturalness × 12 present conditions), and all trials were randomly ordered.

Results: Figure S3 shows the performance for each condition. We performed two-way repeated-measures analysis of variance (ANOVA) for the presenting condition and the naturalness. The main effect of the presenting condition was significant ($F(2, 16) = 13.234$, $p < 0.001$), and the performance under the dynamic condition was the highest for all conditions (multiple comparison test; dynamic condition vs. static condition ($p < 0.005$); dynamic condition vs. shuffle condition ($p < 0.05$)). Not only image information from various viewpoints but also the consecutive images from motion provide us dynamic cues, which supports our hypothesis. The main effect of naturalness was significant ($F(1, 8) = 29.001$, $p < 0.005$) and indicated that the colour inversion decreased the performance of perceptual material discrimination. This is the same tendency as the rotating condition (Figure 2A). There was no

significant interaction ($F(2, 16) = 0.348$, $p = 0.712$). The performance for the colour inversion stimuli was significantly lower than that for the natural stimuli, similar to the upside-down stimuli. These results suggest the luminance polarity of the natural illumination also contributes to the static cue for perceptual material discrimination.

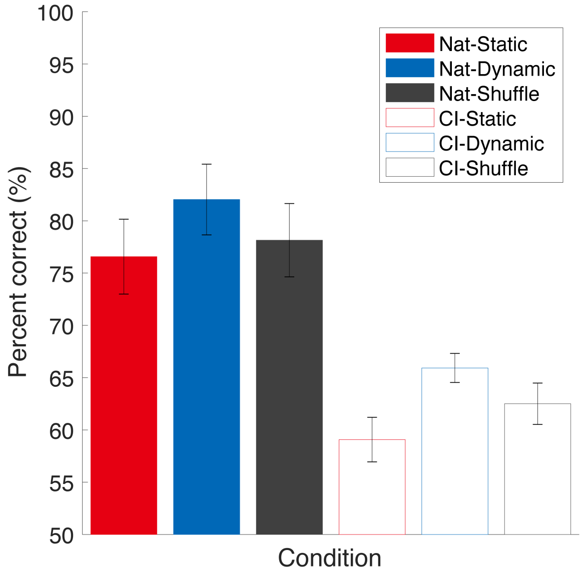

Figure S3. Perceptual material discrimination with the shuffle and colour inversion conditions

The horizontal axis indicates each condition combined with the naturalness and presenting condition. ‘Nat’ signifies natural illumination, and ‘CI’ signifies colour-inverted illumination. The vertical axis indicates the percentage of correct answers. Averages and standard errors among observers were obtained. The error bars represent the standard error of the mean across all nine observers.

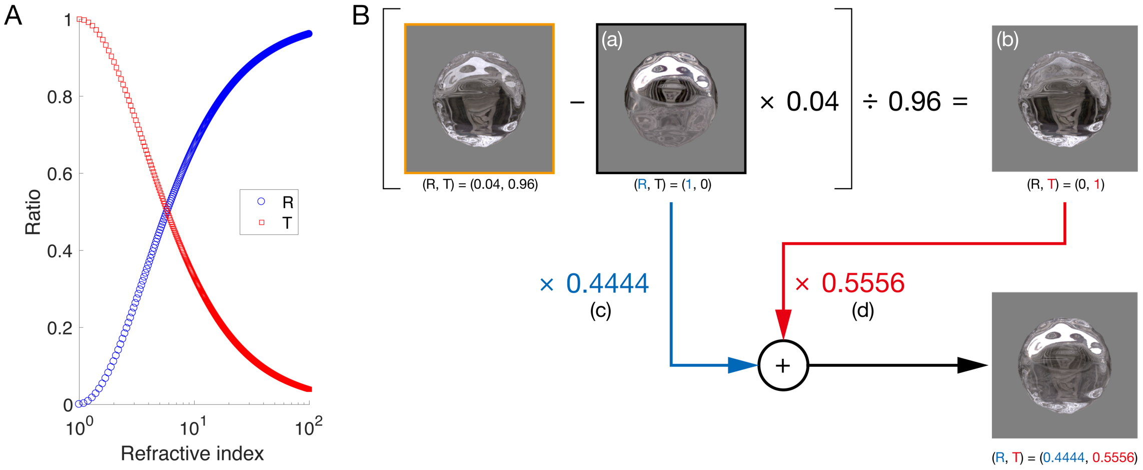

Figure S4. Image morphing method

(A) Characteristics of reflectance and transmittance versus refractive index. The horizontal axis indicates the refractive index. The vertical axis indicates the ratio from 0 to 1. (B) Description of image morphing method. This example shows how to create an image with an arbitrary refractive index of 5. In this case, the reflectance and transmittance are 0.4444 and 0.5556, respectively. The components from (a) to (d) are consistent with the main text.
